# Post-lockdown changes of age-specific susceptibility and its correlation with adherence to social distancing measures

**DOI:** 10.1038/s41598-022-08566-6

**Published:** 2022-03-17

**Authors:** Max S. Y. Lau, Carol Liu, Aaron J. Siegler, Patrick S. Sullivan, Lance A. Waller, Kayoko Shioda, Benjamin A. Lopman

**Affiliations:** 1 Department of Biostatistics and Bioinformatics, Rollins School of Public Health, Emory University, Atlanta, USA; 2 Department of Epidemiology, Rollins School of Public Health, Emory University, Atlanta, USA; 3 Gangarosa Department of Environmental Health, Emory University, Atlanta, USA

**Keywords:** Infectious diseases, Ecological modelling, Epidemiology

## Abstract

Social distancing measures are effective in reducing overall community transmission but much remains unknown about how they have impacted finer-scale dynamics. In particular, much is unknown about how changes of contact patterns and other behaviors including adherence to social distancing, induced by these measures, may have impacted finer-scale transmission dynamics among different age groups. In this paper, we build a stochastic age-specific transmission model to systematically characterize the degree and variation of age-specific transmission dynamics, before and after lifting the lockdown in Georgia, USA. We perform Bayesian (missing-)data-augmentation model inference, leveraging reported age-specific case, seroprevalence and mortality data. We estimate that overall population-level transmissibility was reduced to 41.2% with 95% CI [39%, 43.8%] of the pre-lockdown level in about a week of the announcement of the shelter-in-place order. Although it subsequently increased after the lockdown was lifted, it only bounced back to 62% [58%, 67.2%] of the pre-lockdown level after about a month. We also find that during the lockdown susceptibility to infection increases with age. Specifically, relative to the oldest age group (> 65+), susceptibility for the youngest age group (0–17 years) is 0.13 [0.09, 0.18], and it increases to 0.53 [0.49, 0.59] for 18–44 and 0.75 [0.68, 0.82] for 45–64. More importantly, our results reveal clear changes of age-specific susceptibility (defined as average risk of getting infected during an infectious contact incorporating age-dependent behavioral factors) after the lockdown was lifted, with a trend largely consistent with reported age-specific adherence levels to social distancing and preventive measures. Specifically, the older groups (> 45) (with the highest levels of adherence) appear to have the most significant reductions of susceptibility (e.g., post-lockdown susceptibility reduced to 31.6% [29.3%, 34%] of the estimate before lifting the lockdown for the 6+ group). Finally, we find heterogeneity in case reporting among different age groups, with the lowest rate occurring among the 0–17 group (9.7% [6.4%, 19%]). Our results provide a more fundamental understanding of the impacts of stringent lockdown measures, and finer evidence that other social distancing and preventive measures may be effective in reducing SARS-CoV-2 transmission. These results may be exploited to guide more effective implementations of these measures in many current settings (with low vaccination rate globally and emerging variants) and in future potential outbreaks of novel pathogens.

## Introduction

Social distancing measures ranging from stringent lockdowns to keeping distance are effective in reducing community transmission of SARS-CoV-2^1–3^. Implementing these measures for suppressing SARS-CoV-2 transmission remains an option in many settings, given the current inadequate vaccination rates particularly in low income countries^4^ and uncertainty regarding the level of protection of current vaccines against emerging new variants^5^. It is important to more rigorously quantify the short- to medium-term impacts of these measures on community transmission, particularly for a firmer grasp on the magnitude and timings of the impacts. Moreover, much is unknown about how induced changes in (age-stratified) contact patterns and behaviors by these measures (Ref. Hutchins et al. ^6^) may have impacted finer-scale transmission dynamics—in particular, age-specific transmission dynamics, which are known to be important factors in the transmission of SARS-CoV-2^3,7^. Obtaining systematic characterization of the impacts on both the population-level and finer-scale age-specific dynamics will guide more effective implementations of these social distancing measures in current settings and may also lend insights into future outbreaks of other novel pathogens.

In this paper, we formulate a stochastic transmission modelling framework to systematically characterize population-level and age-specific transmission dynamics amid major changes of lockdown policy in Georgia, USA. Our framework leverages multiple data sources including age-stratified case data of SARS-CoV-2 collected by the Georgia Department of Public Health, and age-stratified contact data and seroprevalence data (see “Study Data”). We perform Bayesian model inference using data-augmentation techniques, which also accounts for unobserved data including unreported cases (see “Materials and Methods”).

## Study data

We leveraged a range of data for this study. Datasets include a large set of COVID-19 age-stratified daily case and mortality data collected by the Georgia Department of Public Health (GDPH), between late March, 2020, and end of June, 2020 (covering the lockdown period and the earliest wave after lifting the lockdown), in the four counties of metro Atlanta (Cobb, DeKalb, Gwinnett, and Fulton) reporting the largest numbers of cases (29,832 reported cases in total). The GDPH Institutional Review Board has previously determined that this analysis is exempt from the requirement for IRB review and approval and informed consent was not required. We also leveraged publicly available social contact data from a representative survey conducted among US adults which include individuals residing in Atlanta^8^. No children less than 18 years were surveyed in our data, we therefore imputed contacts made by individuals aged 0–17 years following Jarvis et al.^9^ (see also Materials and Methods). Age-specific seroprevalence data from a state-wide cross-sectional serosurvey^10–12^, in conjunction with the mortality data, were used to provide important model calibration information (see also “Materials and Methods”).

## Data availability statement

Case data and mortality data are available at https://dph.georgia.gov. Requests of obtaining the seroprevalence data should be made to P. S. Sullivan and A. J. Siegler the PIs of the serosurvey. Computer codes for this paper are available at https://github.com/msylau/Post-lockdown-age-specific-susceptibility-and-its-correlation-with-adherence/.

## Results

### Magnitude and timings of impacts of lockdowns on community transmission

**Figure 1:**
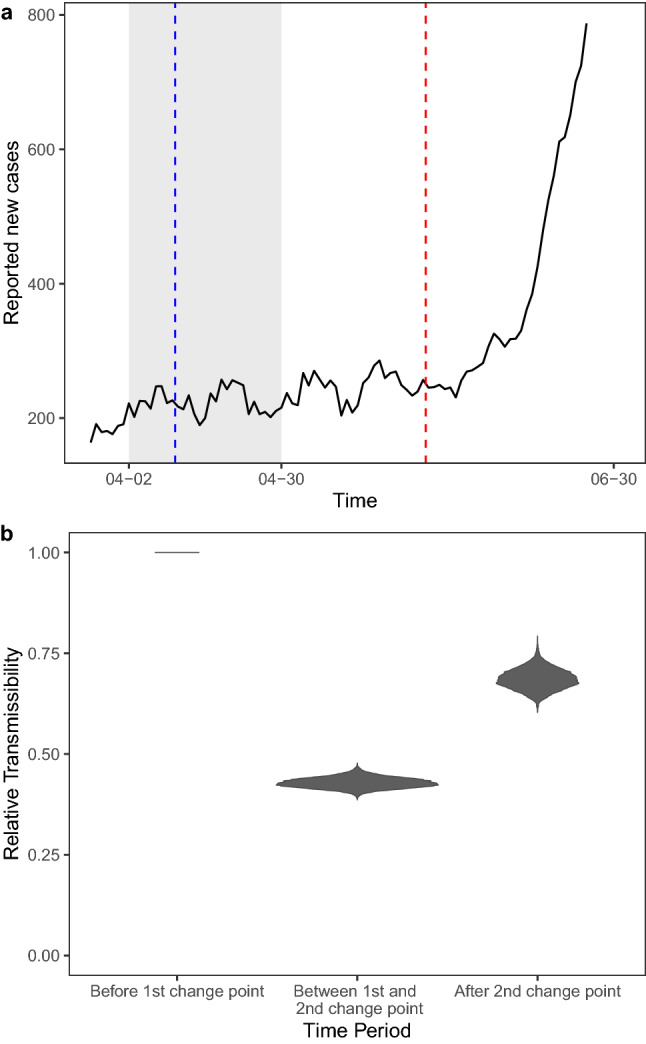
(**a**) Change points. The grey area represents period with active shelter-in-place order. The blue line indicates the model-inferred (free) change point time at which there was a major reduction of population-level transmissibility. The red line indicates the change point at which there was a major uptick of transmissibility. Note that the reduction of transmissibility at first change point also appears to have largely leveled off the overall increasing trend of cases (illustrated by the black curve showing the 7-day moving average) before the next major uptick. (**b**) Transmissibility compared to the transmissibility in the period before the first change point (blue line). It on average reduced to 41.2% [39%, 43.8%] during the period between the first and second change points, and restored to 62% [58%, 67.2%] after the second change point.

Observed new cases often do not directly reflect underlying changes of transmissibility—in particular, a reduction of transmissibility does not manifest in an immediately declining trend of new cases. To more rigorously quantify the impacts of, for example, stringent lockdown measures, it is important to jointly model the magnitude and timings of changes of the underlying transmissibility. Our framework treats these change points as unobserved free model parameters to be estimated from the data. We consider a fixed number change points^3^ but relax prior constraints on the change points (specifically, we used noninformative flat priors for the change points themselves, see also “Materials and Methods”). Our results suggest that population-level transmissibility declined relatively rapidly and substantially after the announcement of shelter-in-place order (see Fig. 1). We estimate that transmissibility was reduced to 41.2% [39%, 43.8%] of the pre-lockdown level in 8.5 days [8.02, 8.97] after the announcement of the shelter-in-place order on April 2, 2020. There also appears to be a carry-over effect of dampening transmission after the order was lifted. Specifically, although the transmissibility subsequently increased after the order was lifted on April 30, it only went back to 62% [58%, 67.2%] before the outbreak peaking again towards the end of our study period.

### Age-specific susceptibility prior to lifting the lockdown

We define *susceptibility* throughout this paper as the average risk of getting infected during an infectious contact. This definition implicitly incorporates potential effects of time- and age-dependent behavioral factors (beyond measures which mostly aim at reducing number of contacts) such as adherence level to facemask wearing which may potentially influence the risk of getting infected (see also Materials and Methods).

**Figure 2:**
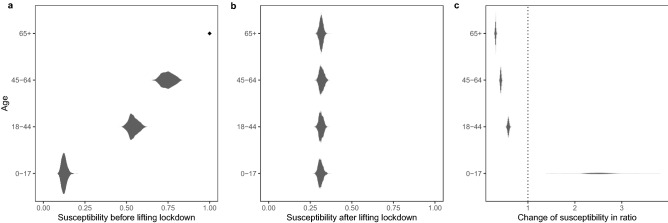
Posterior distributions of age-specific susceptibility. (**a**) Age-specific susceptibility before lifting the lockdown. Note that susceptibility is measured relatively to the 65+ years whose susceptibility parameter is set to be 1. (**b**) Age-specific susceptibility after lifting the lockdown. (**c**) Changes of susceptibility. Change for a particular age group is measured by the ratio between the post-lockdown estimate and the estimate obtained for the period prior to lifting the lockdown.

Susceptibility to SARS-CoV-2 infection was found to be heterogeneous among different age groups. For example, it was estimated that, during earlier phase of the pandemic, susceptibility to infection in individuals under 20 years of age is approximately half that of adults aged 20 years or older^7^. In this paper we considered the following age categories: 0–17 years, 18–44 years, 45–64 years and 65 years and above, and estimate age-specific susceptibility relative to the oldest age group (65+). Our results suggested that, prior to lifting the lockdown, susceptibility for the youngest age group (0–17) is 0.13 [0.09, 0.18], and it increases to 0.53 [0.49, 0.59] for 18–44 and 0.75 [0.68, 0.82] for 45–64 (Fig. 2).

### Post-lockdown age-specific susceptibility

Social contact patterns changed due to the initiation and the subsequent lifting of the shelter-in-place order^8^. Adherence levels to social distancing and preventive measures were also found to vary inhomogeneously among different age groups and in time during our study period^6,13–17^. For example, it was reported in Feehan et al.^8^ that individuals in the younger age groups, compared to those 60+ years, were less likely to follow various measures including mask-wearing, keeping 6 feet distance and avoiding public/crowded places and restaurants. Other surveys also highlight that the discrepancy of adherence between younger and older age groups may only become prominent during the post-lockdown period (e.g., Kim et al.^15^). Such changes and discrepancies may potentially alter the age-specific transmission dynamics, particularly the post-lockdown (i.e., after lockdown was lifted) susceptibility for different age groups.

Our results are largely consistent with these reported trends of age-stratified adherence levels to social distancing and preventive measures. Specifically, the susceptibility of the 0–17 group increases to 2.52 times [1.87, 3.18] the value of its estimate before lifting the order. And, in contrast, Fig. 2 shows that other age groups are all estimated to have reductions of susceptibility, with the trend that the degree of reduction increases with age. In particular, the 65+ age group has the highest degree of reduction of susceptibility among all age groups, being at 31.6% [29.3%, 34%] of its pre-lifting estimate. Also noted that the susceptibility becomes more homogeneous among different age groups.

### Age-specific reporting patterns

**Figure 3:**
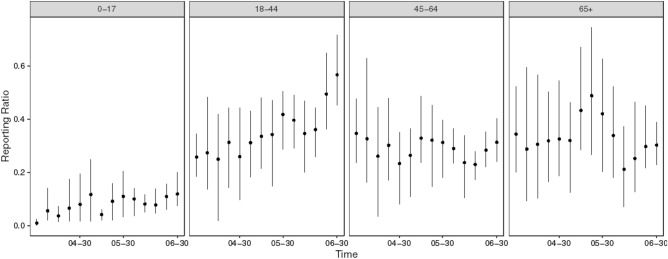
Weekly ratio between inferred reported cases and total cases for different age groups.

Case reporting rates may vary between different age groups due to disparities in severity of symptoms, self-reporting behaviors and testing capacity^7^. Our modelling framework includes and infers an unreported class for each age group (see “Materials and Methods”), from which we are able to estimate age-specific reporting trends. Note that in our framework the unreported class includes asymptomatic cases and any cases which were undetected for other reasons. Figure 3 shows that ratio between inferred reported cases and total cases over time for the 0–17 age group remained the lowest among all age group throughout the study period (9.7% [6.4%, 19%] at the end of our study period). There may be a slight increasing trend of the reporting ratio particularly for those younger than 45, with the estimate for the 18–44 group peaking at 47.3% [44%, 55.5%].

## Model fit

**Figure 4:**
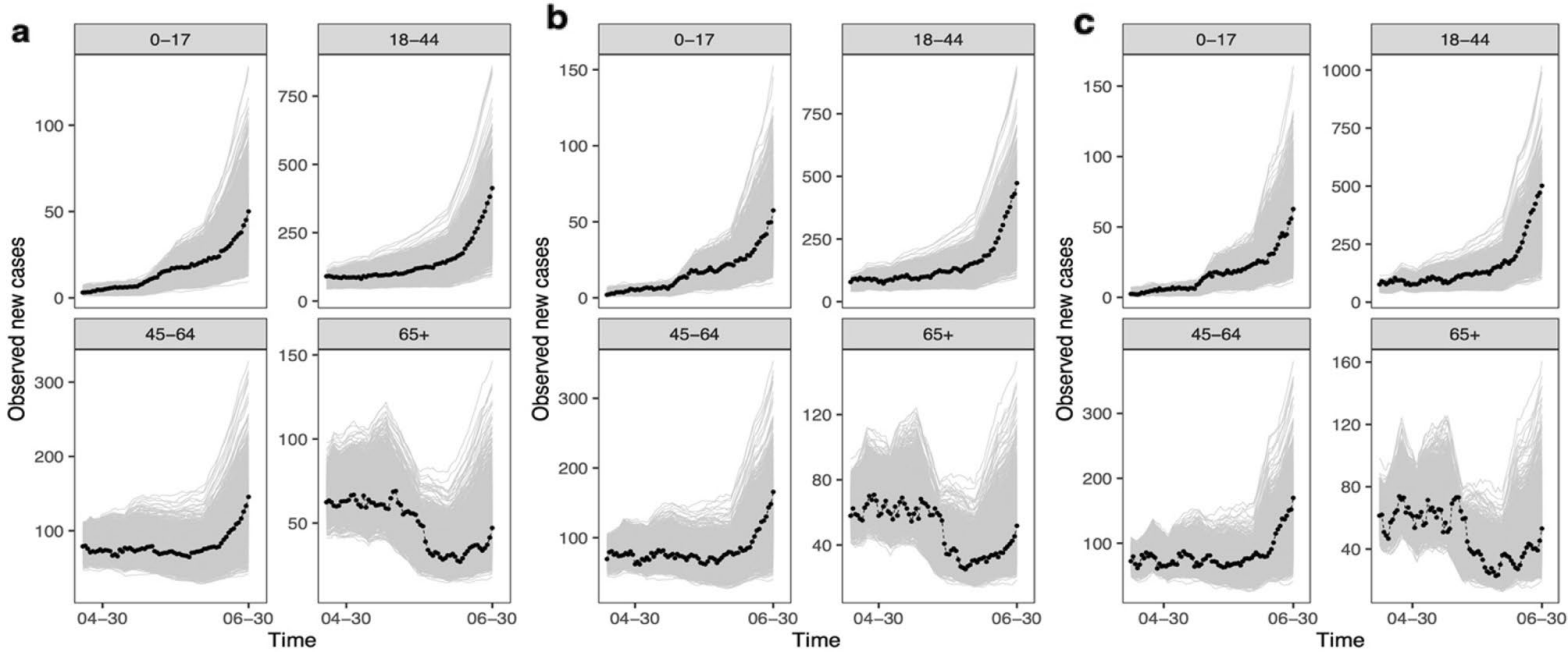
Model fit. (**a**) Daily (14-day moving) average computed from observed daily new cases among different age groups are shown in dotted lines. Grey lines represent the same average computed from 1,000 set of observations simulated from our estimated model. (**b**) Daily (10-day moving) average. (**c**) Daily (7-day moving) average.

To assess the model fit, we compare daily 14-day, 10-day and 7-day moving averages computed from observed new cases and the same metrics computed from data simulated from our estimated model. We simulate the epidemic forward conditional on observations in the first 10 days. Figure 4 shows that our model-simulated data are largely consistent with the observed data. Also note that while the more recent moving average (e.g., 7-day average) for 65+ group is nosier compared to other age groups, our simulated data are capturing the overall trend and size of the outbreak in this group reasonably well. We also explore alternative models with one/three change points. Our results suggest that the one-change-point model does not provide a good model fit (see Figure S1 in SI Figures in Supplementary Information) and the three-change-point model does not yield reliable posterior samples of model parameters.

## Discussion

Our results show that population-level transmissibility declined relatively rapidly after the initiation of shelter-in-place order; and although relaxing the lockdown may be followed by an uptick of cases, underlying population-level transmissibility may stay at a level lower than the level before implementing the lockdown at least within the first two months. These results enable a firmer grasp regarding the short- to medium-term (post-lockdown) impacts of lockdown policies on community transmission of SARS-CoV-2.

Age is an important factor for characterizing transmission dynamics of SARS-CoV-2^3,7^. However, while non-pharmaceutical measures aimed at reducing contacts have shown to be effective in reducing population-wide transmission^18,19^, much is unknown regarding how the age-specific dynamics may have responded to other factors including behavioral changes differed by age induced by the pandemic and lockdown policies. Our results systematically characterize the dynamics of age-specific susceptibility, and provide additional and finer-scale evidence that maintaining social distancing and preventive measures may modify (age-stratified) susceptibility and reduce population-level transmission^1,2^. Specifically, we show that susceptibility of a particular age group may shift amid major changes of lockdown policy, and these shifts appear to correlate with age-specific adherence level to social distancing and preventive measures such as facemask wearing and keeping 6 feet distance. Our results support implementations of these measures in the current setting (with low vaccination rate globally and emerging variants) and in future potential outbreaks of novel pathogens. We also note that as contact patterns tended to be more homogeneous among different age groups during lockdown periods^15^, our estimated differences in age-specific susceptibility occurring in that period may mostly represent biological discrepancies, as opposed to the post-lockdown susceptibility estimates which may largely reflect discrepancy in ‘normal-life’ susceptibility as a combination of both biological and behavioral factors. These age-specific estimates also provide additional insights in framing appropriate and sustainable interventions during the current ‘normal-life’/post-lockdown period. Also note that our results are consistent with Omori et al*.*^20^ which suggest that heterogeneity in age-specific susceptibility may be not sufficient to influence age-specific distribution of mortality which has been largely constant in our study period.

Our study has a number of limitations. First of all, data on adherence levels to social distancing measures are mostly sparse and are not modelled explicitly in our model. Future studies with less sparse joint sampling of adherence data and case data may be considered. Nevertheless, our results show a consistent trend between the changes of susceptibility and adherence levels to social distancing measures reported in other studies. Similarly, while we incorporate data on the number of contacts between age groups, we are not able to dissect potential effects such as types and durations of contacts. Changes in these factors may counteract effects of preventive measures including mask wearing, and it is possible that for those < 18 years the former effect overweighed the latter (e.g., by having longer durations of contacts at schools), resulting the estimated increase in post-lockdown susceptibility (Fig. 2). Future work may need to explicitly model these factors including age-stratified durations and types of contacts, when such data are available including for those < 18 years. Also, future work may include further stratifying the 65+ age group to account for more granular behavioral differences (e.g., the older ones in this group may be more care dependent). Moreover, we have focused on understanding the short- to medium-term impacts of lockdown policies, by focusing on time period that covers both the lockdown period and the neighboring post-lockdown period. Future work may include extending our model and study period. Furthermore, environmental, behavioral and demographic factors are not explicitly incorporated in our transmissibility parameter. For example, while we model time-varying population-level transmissibility, our framework does not explicitly account for potential differences of transmissibility among different age groups (as they are not identifiable with susceptibility parameters). Future data sources such as age-stratified viral load data may be incorporated into our model as future work to dissect age-specific transmissibility from susceptibility. Similarly, other potentially time-varying factors that may impact per-contact transmission probability such as the types and durations of contacts are not explicitly incorporated in our model. Therefore, our estimates should be considered as an average measure of transmissibility aggregating over these various factors. Finally, while our results suggest that the major change points of population-level transmissibility are fairly close to time points of lockdown policy changes (without using strong prior on the change points), other policy and environmental changes may have played a role. For instance, increased efforts for testing-trace-quarantine may have contributed to reduction of population-level transmission. Therefore, our results may represent an aggregate effect of many policies and environmental changes combined, while lockdown related policies and their induced behavioral changes may have been the major contributors.

## Materials and methods

### Stochastic age-specific transmission model

We formulate a stochastic age-specific transmission model in the general Susceptible(S)-Exposed(E)-Reported(I)-Unreported(U)-Recovered(R) framework. For a particular age group $i$ at time $t-1$ ($i=1$ corresponding to the 0–17 years, $i=2$ to 18–44, $i=3$ to 45–64 and $i=4$ to 65+), we have

1 $$\begin{array}{l}{S}_{i}(t)= {S}_{i}(t-1)-{n}_{S{E}_{i}}(t)\\ {E}_{i}(t)= {E}_{i}(t-1)+{n}_{S{E}_{i}}(t)-\\  {n}_{E{I}_{i}}(t)-{n}_{E{U}_{i}}(t)\\ {I}_{i}(t)= {I}_{i}(t-1)+{n}_{E{I}_{i}}(t)-{n}_{I{R}_{i}}(t)\\ {U}_{i}(t)= {U}_{i}(t-1)+{n}_{E{U}_{i}}(t)-{n}_{U{R}_{i}}(t)\\ {R}_{i}(t)= {R}_{i}(t-1)+{n}_{I{R}_{i}}(t)+{n}_{U{R}_{i}}(t),\end{array}$$

where ${n}_{{XY}_{i}}(t)$ represents number of transitions between a class X and class Y for age group $i$ at time $t$.

The number of transitions from the susceptible to exposed class for group $i$ at time $t$ is modelled by

2 $$\begin{aligned}{n}_{S{E}_{i}}(t)&\sim Poi({S}_{i}(t-1)\times {\gamma }_{i}(t)\times \\  & \quad \sum_{j=1}\beta (t)\times {c}_{j,i}(t)\times \{{I}_{j}(t-1)+{U}_{j}(t-1)\}).\end{aligned}$$

Here, $\beta (t)$ denotes the average infectiousness of an infectious individual and ${c}_{j,i}(t)$ is the average number of contacts per day made by age group $j$ to $i$. Also note that the product $\beta (t)\times {c}_{j,i}(t)$ may represent age-specific transmissibility (of age group $j$) accounting for contacts. We allow and infer two change points of $\beta (t)$ (one potentially correlates to changes due to the implementation of lockdown and another one to changes due to the lifting of lockdown), i.e.,

3 $$\beta \left(t\right)=\left\{\begin{array}{ll}{\beta }_{0},&\quad if\; t\le {T}_{1}\\ {\beta }_{1}={\omega }_{1}\times {\beta }_{0},&\quad if \;{T}_{1}<t\le {T}_{2 }\\ {\beta }_{2}={\omega }_{2}\times {\beta }_{0},&\quad if\; t>{T}_{2},\end{array}\right.$$

where ${T}_{1}$ and ${T}_{2}$ are the two change points to be inferred (${T}_{2}\ge {T}_{1}$). ${\gamma }_{i}(t)$ denotes the susceptibility of group $i$ relative to the oldest age group (i.e., ${\gamma }_{4}=1$), which is also allowed to change proportionally after lifting the lockdown. Note that ${\gamma }_{i}(t)$ implicitly incorporates any behavioral effects (e.g., potential reduction of risk of getting infection due to facemask wearing). Transitions between other classes are modelled as:

4 $$\begin{aligned}{n}_{E{U}_{i}}(t)\sim & Bin({n}_{S{E}_{i}}(t-{D}_{EU}),{p}_{{U}_{i}}(t-{D}_{EU}))\\ {n}_{E{I}_{i}}(t)=& {n}_{S{E}_{i}}(t-{D}_{EI})-{n}_{E{U}_{i}}(t)\\ {n}_{I{R}_{i}}(t)=& {n}_{E{I}_{i}}(t-{D}_{IR})\\ {n}_{U{R}_{i}}(t)=& {n}_{E{U}_{i}}(t-{D}_{UR}),\end{aligned}$$

where ${D}_{EI}$, ${D}_{EU}$, ${D}_{IR}$ and ${D}_{UR}$ denote the mean waiting times between the indicated two classes. We assume that ${D}_{EI}$= ${D}_{EU}$=7 days and ${D}_{IR}$= ${D}_{UR}$=14 days. ${p}_{{U}_{i}}(t)$ represents probability that an infection is unreported at times $t$ for age group $i$, we assume

5 $${p}_{{U}_{i}}(t)=1-\frac{{e}^{{f}_{i}(t)}}{1+{e}^{{f}_{i}(t)}}.$$

**Figure 5:**
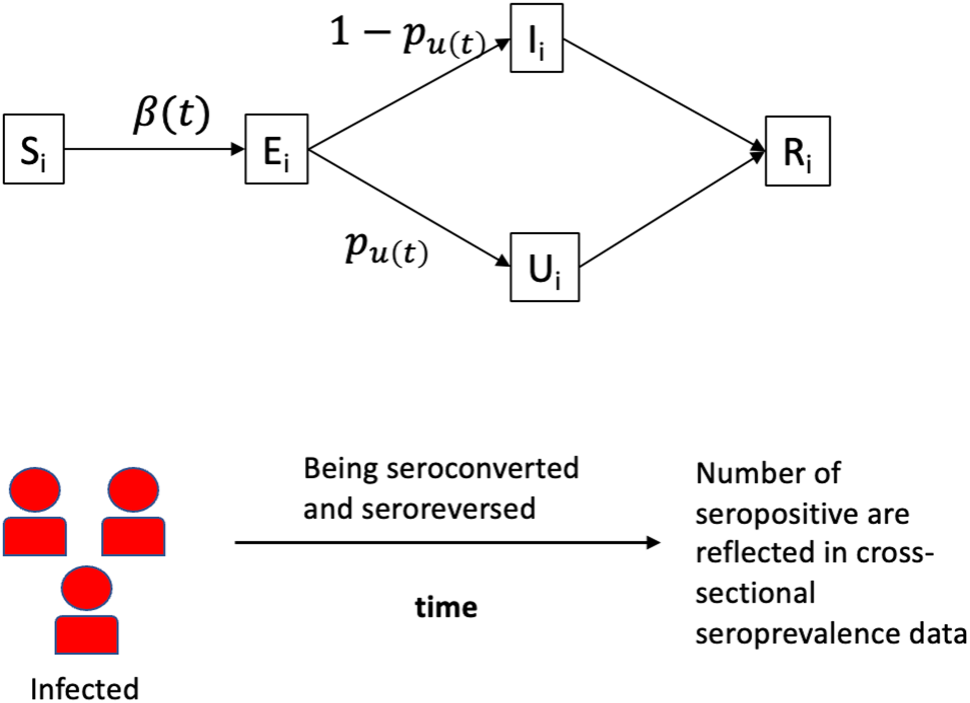
A schematic illustration of our modelling framework.

${f}_{i}(.)$ is an increasing function with ${f}_{i}(t)={a}_{i}+{b}_{i}\times t$, where $-\infty <{a}_{i}<\infty$ and ${b}_{i}\ge 0$, which is used to model time-varying average reporting rate in a particular age group $i$ (which may be increasing due to, for example, increasing efforts for asymptomatic screening and testing). We provide a schematic overview of our modelling framework in Fig. 5.

We also explore the sensitivity of the assumption ${D}_{UR}$= ${D}_{IR}$. Specifically, we also consider the scenario when ${D}_{UR}$= ${0.5\times D}_{IR}$ . Our results show that our main conclusions are largely robust towards the assumption (see Table S1 in SI). In particular, the trend of susceptibility increasing with age (prior to lifting the lockdown) and the homogeneity of susceptibility after lifting the lockdown remain robust. However, we do observe that transmissibility is estimated to be higher in the scenario ${D}_{UR}$= ${0.5\times D}_{IR}$, but maintaining the same trend obtained under the assumption of ${D}_{UR}$= ${D}_{IR}$.

### Bayesian model inference and data-augmentation

We infer ${\varvec{\Theta}}$ (i.e. the parameter vector) in the Bayesian framework by sampling it from the posterior distribution $P\left({\varvec{\Theta}}|\mathbf{z}\right)$ where $\mathbf{z}$ include both observed and unobserved data^21–24^. Denoting the likelihood by $L({\varvec{\Theta}};\mathbf{z})$, the posterior distribution of ${\varvec{\Theta}}$ is $P\left({\varvec{\Theta}}|\mathbf{z}\right))\propto L\left({\varvec{\Theta}};\mathbf{z}\right)\pi \left({\varvec{\Theta}}\right)$, where $\pi ({\varvec{\Theta}})$ is prior distribution for ${\varvec{\Theta}}$. Markov chain Monte Carlo (MCMC) techniques are used to obtain samples from the posterior distribution. We assumed that, at time 0, the ratio between observed and unreported cases was assumed to be 1/10^25^. Mortality and seroprevalence data are used to facilitate the estimation of the number of recovered individuals ${R}_{i}(t)$. Specifically, knowing that number of recovered is bounded above by the cumulative incidence, prior distribution of the number of recovered individuals ${R}_{i}(t)$ was assumed to follow a Uniform distribution bounded above by the cumulative incidence. The cumulative incidence is estimated from the mortality data and cross-sectional seroprevalence data following the approach previously developed by the authors^26^. As seroprevalence data were not collected for the 0–17 age group, we conservatively assume that ${R}_{i=1}(t)$ was bounded above by the estimated cumulative incidence of the 18–44 group. Non-informative uniform priors for parameters in ${\varvec{\Theta}}$ are used (see *Supplementary Information (SI)*). More details of the inferential algorithm are referred to *SI Text in Supplementary Information (SI)*. Posterior distributions of parameters are given in Figure S2 in *SI Figures*.

### Imputations of missing contacts

Since no children younger than 18 years were surveyed in our data, we imputed (during-pandemic) contacts made by individuals aged 0–17 years. Specifically, following Jarvis et al.^9^, we take pre-pandemic contacts and rescale them based on the ratio of the dominant eigenvalue of a during-pandemic matrix to dominant eigenvalue of the pre-pandemic matrix. Also, since there are no publicly available pre-pandemic (0–17 years) contact data for the US, we used pre-pandemic estimates from the UK POLYMOD study as a proxy in the imputation^27,28^.

## Supplementary Information

Supplementary Information.
