# Supplementary material for: Post-lockdown changes of age-specific susceptibility and its correlation with adherence to social distancing measures
Source: Sci Rep. 2022 Mar 17;12:4637. doi: 10.1038/s41598-022-08566-6 (PMC8929451; doi:10.1038/s41598-022-08566-6)
Supplement: Supplementary file 1 — Supplementary Information. [file 41598_2022_8566_MOESM1_ESM.pdf]

Post-lockdown changes of age-specific susceptibility and its correlation with adherence to social distancing measures

Max SY Lau [a,*], Carol Liu [b], Aaron J Siegler [b], Patrick S Sullivan [b], Lance A Waller [a], Kayoko Shioda [b, c], and Benjamin A Lopman [b]

[a]Department of Biostatistics and Bioinformatics, Rollins School of Public Health, Emory University; [b]Department of Epidemiology, Rollins School of Public Health, Emory University; [c]Gangarosa Department of Environmental Health, Emory University

**Corresponding author*

Supplementary Information (SI)

SI Text

Statistical Inference and Data-augmentation. We conduct Bayesian inference of the partially observed outbreak using the process of data augmentation supported by Markov chain Monte Carlo methods[21,22,23]. Let $\mathbf{\Theta} = (\beta(t), \gamma_i(t), \omega_1, \omega_2, T_1, T_2, a_i, b_i)$ for $i = 1,2,3,4$. Given observed partial data $\mathbf{y}$ (e.g., here we do not observe unreported cases), the inference involves sampling from the joint posterior distribution $\pi(\mathbf{\Theta}, \mathbf{z}|\mathbf{y}) \propto L(\mathbf{\Theta}; \mathbf{z})\pi(\mathbf{\Theta})$, where $\mathbf{z}$ represents the complete data and $\pi(\mathbf{\Theta})$ represents the prior distribution of model quantities, such that the complete $\mathbf{z}$ is reconstructed, or 'imputed'. Note that the complete likelihood function is given by Equation (4) in the main text.

Parameters in $\mathbf{\Theta}$ are updated sequentially with a standard random-walk Metropolis-Hastings algorithm[24]. For example, a new parameter value $\omega_1{}'$ is proposed from a normal distribution centered on the current value of ω_1

$$\omega_1{}' \sim \omega_1 + N(0, \rho^2)$$

where ρ controls the step-size of the random-walk. Age-specific susceptibility $\gamma(t)_i$s are highly correlated among age groups and to improve mixing, we use the Metropolis-Hastings algorithm with blockings for these two parameters. They are drawn using multivariate Normal distribution as proposal distributions, with the mean values taken to be current parameter values (i.e., a random walk), and with entries in the covariance matrix estimated from the samples generated from a preliminary run of the unblocked version of the MCMC algorithm (i.e., each parameter is sampled sequentially using random-walk Metropolis-Hastings). We used improper flat non-informative priors for all parameters in $\mathbf{\Theta}$.

The number of newly infected for age group i at time t $n_{SE_i}(t)$ was updated using a Metropolis-Hastings algorithm with discrete random-walk, i.e.

$$n_{SE_i}{}'(t) = n_{SE_i}(t) + U[-10,10],$$

and $n_{SE_i}(t)$ was bounded between the reported new cases $n_{EI_i}(t + D_{EI})$ and the number of susceptible $S_i(t-1)$. We also updated $R_i(t)$ the initial number of individuals in class R for each age group, using a similar algorithm in updating $n_{SE_i}(t)$ but imposing an upper bound at the estimated cumulative incidence for a particular age group using an approach previously developed by the authors[26]. Briefly, a Bayesian model was developed to estimate the infection fatality ratio (IFR) using age-specific mortality data and population-level cross-sectional seroprevalence data, accounting simultaneously for

seroconversion (acquisition of the detectable level of antibodies) and seroreversion (loss of detectable antibodies). The estimated IFR then can used to calculate the cumulative incidence − given that total number of infections on day k can be computed as the number of reported deaths divided by the estimated IFR. Full details of this approach are detailed in the previously published paper[25]. The upper bound of the prior of $R_i(t)$ is then conservatively assumed to be the upper limit of the 95% CI of the estimated cumulative incidence at time t.

SI Figures

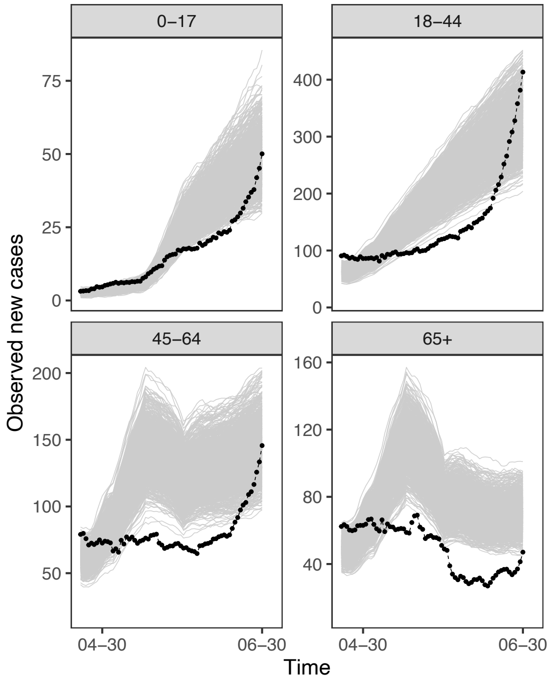

Fig. S1. Model fit of the model with one change point. Daily (14-day moving) average computed from observed daily new cases among different age groups are shown in dotted lines. Grey lines represent the same average computed from 1,000 set of observations simulated from the estimated model.

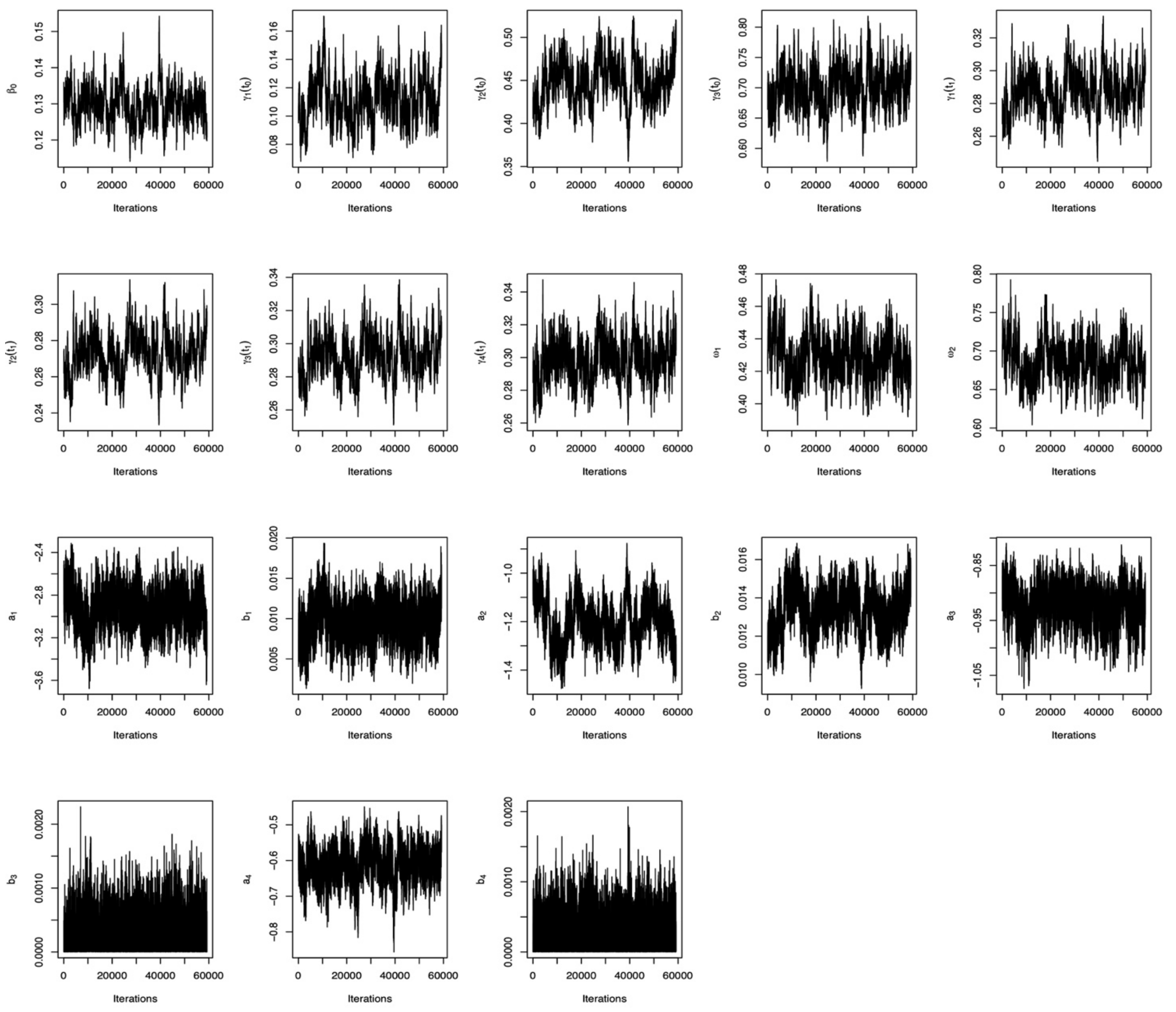

Fig. S2. MCMC outputs of our model inference with a thinning interval 40 iterations (we ran the chains for 2,500,000 iterations and used a burn-in 100,000 iterations). All chains appear to converge by visual inspection and they also pass convergence tests (i.e., Heidelberger and Welch's diagnostic in the R package coda).

SI Tables

Parameters \ Assumptions	D_{UR}= D_{IR}	D_{UR}= 0.5 × D_{IR}
Susceptibility before lifting lockdown for 0-17	0.13 [0.09, 0.18]	0.1 [0.07, 0.13]
Susceptibility before lifting lockdown for 18-44	0.53 [0.49, 0.59]	0.31 [0.27, 0.43]
Susceptibility before lifting lockdown for 45-64	0.75 [0.68, 0.82]	0.79 [0.74, 0.9]
Susceptibility after lifting lockdown for 0-17	0.29 [0.26, 0.31]	0.25 [0.23, 0.3]
Susceptibility after lifting lockdown for 18-44	0.27 [0.25, 0.3]	0.23 [0.21, 0.29]
Susceptibility after lifting lockdown for 45-64	0.29 [0.27, 0.32]	0.25 [0.23, 0.3]
Susceptibility after lifting lockdown for 65+	0.29 [0.27, 0.32]	0.27 [0.25, 0.31]
Magnitude of transmissibility compared to pre-lockdown level at first change point	41.2% [39%, 43.8%]	48% [46%, 52%]
Magnitude of transmissibility compared to pre-lockdown level at second change point	62% [58%, 67.2%]	75% [71%, 83%]

Table S1. Sensitivity analysis testing the robustness of the assumption of D_{UR}= D_{IR}.
